# HS–GC–IMS Coupled With Chemometrics Analyzes Volatile Aroma Compounds in Steamed *Polygonatum cyrtonema* Hua at Different Production Stages

**DOI:** 10.1155/jamc/5592877

**Published:** 2025-03-10

**Authors:** Bingbing Shen, Rongrong Zhou, Jia Lao, Jian Jin, Wei He, Xin Zhou, Hao Liu, Jing Xie, Shuihan Zhang, Can Zhong

**Affiliations:** ^1^Institute of Chinese Medicine Resources, Hunan Academy of Chinese Medicine, Changsha 410013, China; ^2^Department of Research and Development, Resgreen Group International Inc., Changsha 410329, China; ^3^Graduate School, Hunan University of Chinese Medicine, Changsha 410208, China

**Keywords:** chemometrics, HS–GC–IMS, nine-steam-nine-bask, *Polygonatum cyrtonema* Hua, volatile aroma compounds

## Abstract

Headspace-gas chromatography-ion migration spectrometry (HS–GC–IMS) combined with chemometrics was used to analyze the changes in volatile aroma compounds (VOCs) at different production stages of steaming *Polygonatum cyrtonema* Hua. Fifty-seven representative compounds in the process of steaming were identified, including 17 aldehydes, 15 alcohols, 15 ketones, 5 esters, 3 furans, and 2 acids. After steaming, the content of 21 compounds decreased. Among them, 3 compounds gradually decreased along with an increase in steaming times; they were 1-hexanol dimer, 1-hexanol monomer, and 3-methylbutan-1-ol dimer. The content of 14 compounds increased than before, and that of three, 1-(2-furanyl)ethanone monomer, 2-furaldehyde, and 3-methyl butanal, increased significantly in the steaming times. The VOCs of the different samples can be classified by GC–IMS data combined with principal component analysis (PCA) and heatmap cluster analysis. A reliable prediction set was established by orthogonal partial least squares discriminant analysis (OPLS-DA), and 18 different VOCs with projected variable importance (VIP) greater than 1.0 were screened out, which could be used as differentiating markers. Therefore, HS–GC–IMS and PCA were used to rapidly identify and classify the VOCs in different production stages of steaming *P. cyrtonema* Hua.

## 1. Introduction

*Polygonatum cyrtonema* Hua is a valuable source of Polygonati Rhizoma, with both medicinal and functional food [1]. It was first published as a top-grade herb in “*Ming Yi Bie Lu*” in China. Modern studies have found that *polygonatum* possesses polysaccharides, flavonoids, saponins, and other constituents [2–4] that have proven pharmacological effects such as lowering blood sugar, regulating blood lipids, lowering blood pressure, antitumor, antifatigue, and other pharmacological effects [5–8].

Since ancient times, the processing methods of *P. cyrtonema* Hua mainly include steaming, wine steaming, nine-steam-nine-bask, and black bean processing [9, 10]. Among them, the processing method of nine-steam-nine-bask, which involves repeated steaming and drying, is the most widely used and has the longest history [11]. After nine times of steaming and sun drying, the color becomes black, and the taste changes to sweet. The chemical constituents of *P. cyrtonema* Hua also change quietly, the tonic effect is enhanced, and the irritation of the throat disappears [12, 13]. So far, a few have reported changes in volatile aroma compounds during the process of steaming.

Headspace-gas chromatography-ion mobility spectrometry (HS–GC–IMS) is a new method for the detection of volatile flavor compounds (VOCs) in solid or liquid samples. It combines the advantages of high separation through GC and the high sensitivity of IMS to quickly detect trace amounts of VOCs in samples without any special pretreatment [14, 15]. It is widely used in odor detection in Traditional Chinese Medicine and clinical, food, and environmental analysis [16–19]. Among them, Traditional Chinese Medicine is mainly used in the identification of different varieties of TCM, pre- and postprocessing analysis, comparison of different processed stages, different drying methods, and so on [12, 20, 21].

The study of VOCs in *P. cyrtonema* Hua is important due to its culinary significance and potential health benefits, as these compounds may correlate with the herb's medicinal properties. In addition, understanding its aroma can enhance quality control. Finally, this research contributes to the broader field of aroma analysis, utilizing advanced techniques to uncover valuable insights into the herb's chemical composition. So we used HS–GC–IMS technology to rapidly analyze and identify the VOCs in different production stages of steaming to provide a theoretical basis for the processing, production, and quality control of *P. cyrtonema* Hua.

## 2. Methods and Materials

### 2.1. Materials

*P. cyrtonema* Hua (wet) was obtained from Anhua (Hunan, China). Samples were steamed and dried nine times in the laboratory, and the processed samples were stored in Room 312 on the third floor of the laboratory building.

### 2.2. The Different Production Stages of Steaming *Polygonatum cyrtonema* Hua

The processing method of *P. cyrtonema* Hua refers to the previously published articles [22]. The whole dried rhizome of *P. cyrtonema* Hua was steamed for 2 h at 105°C and then placed for 22 h in a high-pressure steam sterilization pot (0.12 MPa) with 1–9 cycles. The fresh sample was marked as PF and the processed *P. cyrtonema* Hua samples from one, two, three, four, five, six, seven, eight, and nine cycles were collected and marked as P1, P2, P3, P4, P5, P6, P7, P8, and P9, respectively.

### 2.3. HS–GC–IMS Analysis

In this experiment, the IMS instrument (FlavourSpec, Gesellschaft für Analytische Sensorsysteme mbH, Dortmund) equipped with a CTC-PAL 3 static headspace automatic sampling device (CTC Analytics AG) that can be directly sampled from the headspace using a 1 mL air-tight heated syringe was used. The GC Agilent 490 (Agilent Technologies) was operated using the carrier gas nitrogen (99.999% purity) at programmed flow rates using a MXT-WAX capillary column (30 m, 0.53 mm ID, 1 μm FT) at a constant temperature of 60°C. Each sample was accurately weighed at 1.0 g and placed in a 20 mL headspace bottle, and incubated at 80°C for 20 min before sampling.

#### 2.3.1. Conditions of the Headspace Autosampler

The automatic sampler was set at 80°C incubation temperature for 20 min and the speed of 500 rpm. The injection needle had a temperature of 85°C and an injection volume of 500 μL without a shunt.

#### 2.3.2. Conditions of Gas Chromatography and Ion Mobility Spectrometry

The samples were driven into a MXT-WAX capillary column (60°C) with a nitrogen flow of 150 mL/min at a programmed flow of 2 mL/min for 2 min, 10 mL/min for 8 min, 100 mL/min for 10 min, and 150 mL/min for 30 min. Then, the analytes were eluted and ionized in the IMS ionization chamber. The resulting ions were driven into a 98 mm long drift tube which operated at a constant temperature (45°C) and voltage (5 kV).

### 2.4. Statistical Analysis

The identification of VOCs was based on the retention index (RI) and drift time (reactive ion peak [RIP] relative) in the GC–IMS library. The analysis software used was VOCal and three plugins (Reporter, Gallery Plot, and Dynamic principal component analysis (PCA) plugins). VOCal software was used to view the analytical spectrum and qualitatively and quantitatively analyze the data. The Reporter plugin can directly compare the differences in spectra of the samples (3D spectrum, 2D top view, and difference spectrum). The Gallery Plot plugin for fingerprint comparison was used to compare the differences in VOCs intuitively and quantitatively among different samples. Chemometrics were performed by SIMCA 14.1 (MKS Umetrics, Sweden).

## 3. Results and Discussion

### 3.1. Color and Odor Assessments

**Figure 1:**
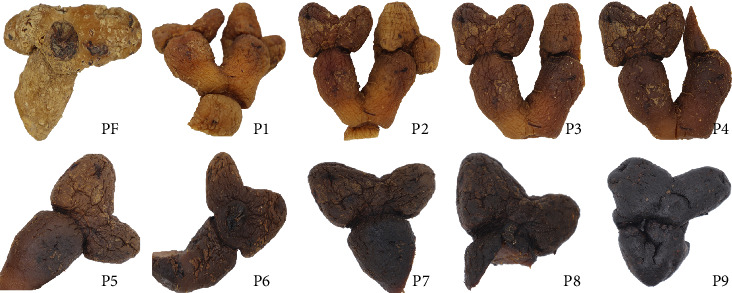
The picture of different production stages of steaming *Polygonatum cyrtonema* Hua.

The appearance of *P. cyrtonema* Hua during the nine-steam-nine-bask processing is shown in Figure 1. The fresh *P. cyrtonema* Hua (PF) sample was bright yellow, as expected. With the increase in processing time, the color gradually darkened, and the change was inconspicuous after three to four processing cycles. Since then, the color remained dark brown; meanwhile, the odor intensity changed proportionally during processing. The distribution of the fresh from the other steaming samples could be clearly observed. The essence was divided into four categories based on color and odor: PF, Step 1 (P1, P2, and P3), Step 2 (P4, P5, and P6), and Step 3 (P7, P8, and P9). In some reports, thermal processing often changed the color of the sample and affected the quality of obtained products [22]; the changes were associated with the Maillard reaction. During the nine-steam-nine-bask processing, the reducing sugar of *P. cyrtonema* Hua reacted with amino compounds to produce brown or even black macromolecular substances. However, the formation and conversion of characteristic volatile compounds in the processing step need further analysis. In this study, PF, P3, P6, and P9 were investigated via GC–IMS to find the relationship between the final flavor and the entire nine-steam-nine-bask processing.

### 3.2. Analysis of Different Production Stages of Steaming *P. cyrtonema* Hua by HS–GC–IMS

**Figure 2:**
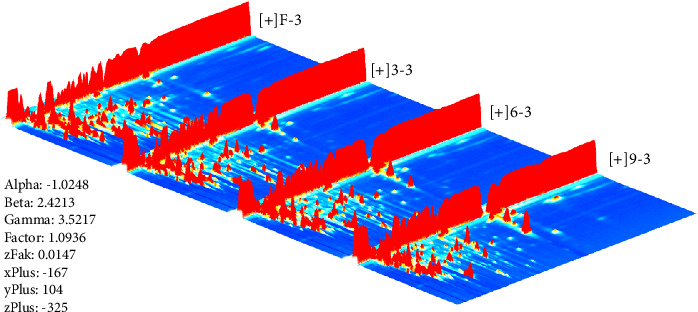
The 3D spectrum of different production stages of steaming *Polygonatum cyrtonema* Hua (PF, P3, P6, and P9) analyzed by headspace-gas chromatography-ion mobility spectrometry.

In this study, the VOCs in different production stages were determined by HS–GC–IMS. The HS-GS-IMS 3D spectrum of PF, P3, P6, and P9 are presented in Figure 2. The *X*-, *Y*-, and *Z*-axes represented ion drift time, retention of GC, and ion peak strength, respectively [23]. From this 3D drawing, we can intuitively observe the difference in different production stages of steaming *P. cyrtonema* Hua (PF, P3, P6, and P9). The background is blue, and each signal spot to the right of the RIP represents a volatile compound. For better observation, in this study, a top view was used for comparative analysis of differences.

The top view plot (Figure S1-1) of HS–GC–IMS was obtained by the normalization of the ion drift time and RIP position. The whole spectrum represents the total VOCs, and each color indicates the concentration of individual compounds. White indicates a lower concentration and red indicates a higher concentration; the darker the color, the greater the concentration. Most signals appeared at a 100–1500(s) retention time and 1.0–1.75 drift time.

To clearly compare different samples, different comparison models were used. In this model, the PF spectrum was selected as the reference, and the spectra of other samples were subtracted (Figure S1-2). If the VOCs were the same, the background after deduction was white, while red indicated a higher concentration of the compound than the reference, and blue indicated a lower concentration of the compound than the reference. The red area is mainly distributed between 1100–1500(s) and 1.0–1.25, while the blue area is distributed between 500–1000(s) and 1.5–1.75.

### 3.3. Fingerprint Analysis of Different Production Stages of Steaming *P. cyrtonema* Hua

Fingerprints were used to further identify specific differences in different production stages. In the fingerprint, each row represents the signal peak of all compounds in one kind of sample, while each column represents the signal peak of the same volatile compound in different samples. The brightness of each square roughly represents the content of the volatile compound. Ninety-two compounds were produced in different processing stages of steaming *P. cyrtonema* Hua, of which 57 were identified (Table 1). As shown in Figure S2, the components included 17 aldehydes, 15 alcohols, 15 ketones, 5 esters, 3 furans, and 2 acids. The percentage of each type of compound is shown in Figure S3. Here, the contents of alcohol and ester were the highest in PF samples than in others and decreased with the progress in the production stages. Among them, 1-hexanol and 1-pentanol were present in three forms, as monomers, dimers, and polymers. There were 15 kinds of compounds with two existing forms, of monomers and dimers, mainly including (E)-2-octenal, (E)-2-heptenal, (E)-2-hexenal, 1-(2-furanyl)ethenone, 1-butanol, 1-hydroxy-2-propanone, 2-methyl-1-propanol, 2-methyltetrahydrofuran-3-one, 3-hydroxy-2-butanone, 3-methylbutan-1-ol, 4-methyl-2-pentanone, acetic acid, cyclopentanone, ethyl hexanoate, and heptaldehyde.

**Figure 3:**
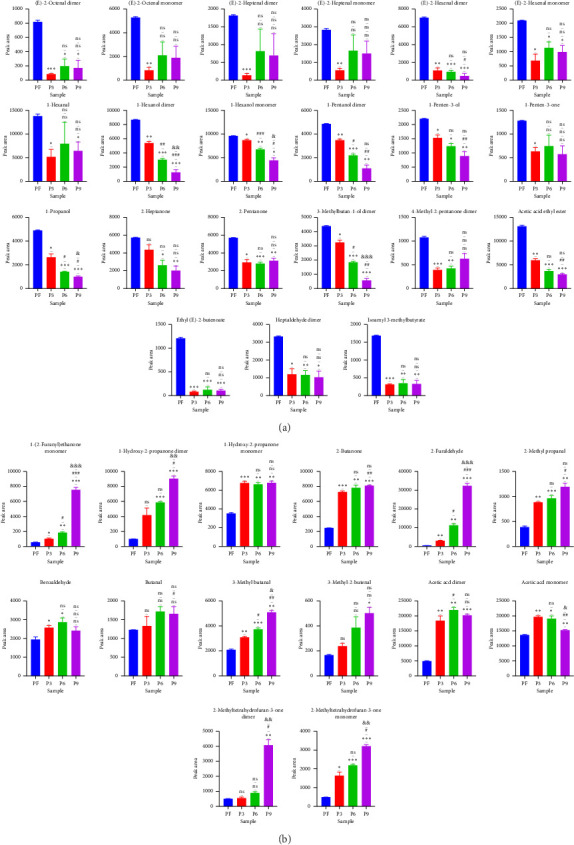
(a) The column chart showing that the concentration of compounds in Region A before steaming was higher than after (all values are expressed as the mean ± standard deviation of three independent experiments; ⁣^∗^*p* < 0.05, ⁣^∗∗^*p* < 0.01, and ⁣^∗∗∗^*p* < 0.001 compared to PF; ^#^*p* < 0.05, ^##^*p* < 0.01, and ^###^*p* < 0.001 compared to P3; ^&^*p* < 0.05, ^&&^*p* < 0.01, and ^&&&^*p* < 0.001 compared to P6; ns *p* > 0.05). (b) The column chart of the concentration of compounds in Region B after steaming is higher than before (all values are expressed as the mean ± standard deviation of three independent experiments; ⁣^∗^*p* < 0.05, ⁣^∗∗^*p* < 0.01, and ⁣^∗∗∗^*p* < 0.001 compared to PF; ^#^*p* < 0.05, ^##^*p* < 0.01, and ^###^*p* < 0.001 compared to P3; ^&^*p* < 0.05, ^&&^*p* < 0.01, and ^&&&^*p* < 0.001 compared to P6; ns *p* > 0.05).

**Figure 4:**
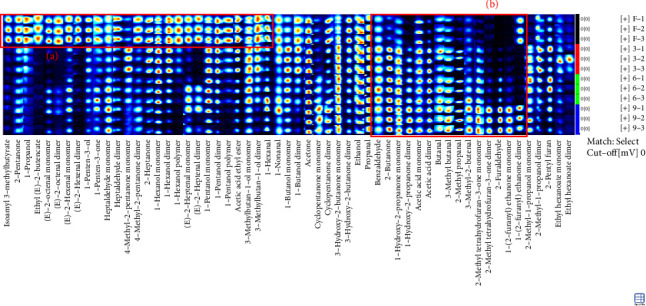
The Gallery Plot of volatile compounds in different processing stages of steaming *Polygonatum cyrtonema* Hua.

Further comparison of the changes before (PF) and after (P3, P6, and P9) steaming revealed common regions in the samples and exhibited their characteristic peaks. As shown in Figure 3, in PF samples, compounds in Region A had the highest concentration and were mainly isoamyl 3-methylbutyrate, 2-pentanone, 1-propanol, ethyl (E)-2-butenoate, (E)-2-octenal, (E)-2-hexenal, 1-pentene-3-ol, 1-pentene-3-one, heptaldehyde, 4-methyl-2-pentanone, 2-heptanone, 1-hexanol, (E)-2-heptenal, 1-pentanol, acetic acid ethyl ester, 3-methylbutan-1-ol, and 1-hexanol (Figure 4(a)). In addition, with the increase in the number of processing times, the compounds whose concentration decreased gradually included 1-hexanol dimer(**12**), 1-hexanol monomer(**13**), and 3-methylbutan-1-ol dimer(**38**) (*p* < 0.05). On the other hand, in Region B, the concentrations of VOCs after steaming were higher than before steaming and included benzaldehyde, 2-butanone, 1-hydroxy-2-propanone, acetic acid, butanal, 3-methyl butanal, 2-methyl propanal, 3-methyl-2-butenal, 2-methyltetrahydrofuran-3-one, 2-furaldehyde, and 1-(2-furanyl)ethenone (Figure 4(b)). With the increase in steaming times, the compounds whose concentration increased significantly included 1-(2-furanyl)ethanone monomer(**8**), 2-furaldehyde(**25**), and 3-methyl butanal(**36**) (*p* < 0.05).

### 3.4. Resemblance Analysis of Different Production Stages of Steaming *P. cyrtonema* Hua by PCA and Partial Least Squares Discrimination Analysis (PLS-DA)

PCA is a common statistical method often used to show the differences between samples [24]. PCA of different production stages of steaming *P. cyrtonema* Hua is shown in Figure S4. The parallel samples of different processed products appear closely spaced, indicating a good parallelism. The distance between fresh products and steamed samples is far, indicating a great difference in volatile components before and after steaming. The distance between samples with different times of steaming was shorter, indicating a little difference between different times of steaming. To sum up, the conclusion of PCA is consistent with that of Gallery Plot, and PCA analysis could distinguish the four samples. In addition, the contribution rates of principal components PC1 and PC2 were 59.8% and 29.3%, respectively.

In order to further understand the changes of VOCs during processing, 57 VOCs were used as independent variables and their different steaming times' samples were used as dependent variables to perform PLS-DA. As shown in Figure S5 (*X* [1] = 0.658, *X* [2] = 0.256), the PF samples were on the rightmost of the Figure S5 and P9 were on the leftmost. The results indicate that steaming is the key factor in VOC change.

### 3.5. Differences of VOCs at the Different Production Stages of Steaming *P. cyrtonema* Hua

To clarify the differences of VOCs at the different production stages of steaming *P. cyrtonema* Hua, orthogonal PLS-DA (OPLS-DA) of PF vs. P3 and P3 vs. P6 and P6 vs. P9 was performed. OPLS-DA is a supervised statistical method for discriminant analysis that could better access information from group differences. Through analysis, a projected variable importance (VIP) value can be obtained for every metabolite, and the larger the VIP value, the greater the contribution to distinguish the two groups [25]. The OPLS-DA of PF vs. P3 are shown in Figure S6-1a (R_2_X [cum] = 0.981, R_2_Y [cum] = 1.0, *Q*2 = 1), Figure S6-2a (R_2_X [cum] = 0.842, R_2_Y [cum] = 0.999, *Q*2 = 0.990), and Figure S6-3a (R_2_X [cum] = 0.884, R_2_Y [cum] = 0.998, *Q*2 = 0.988), which showed that different production stages of steaming *P. cyrtonema* Hua might be classified through OPLS-DA. In order to prevent overestimation, the accuracy of the OPLS-DA model was confirmed through a rearrangement test, and the results of 200 transverifications showed that R2 and Q2 of all rearrangement trials were lesser than the raw data, and Q_2_Y is all worth less than 0.05, indicating that the simulation equation was not overadapting (Figures S6-1b, S6-2b, and S6-3b). Therefore, the established OPLS-DA simulation results are consistent and reliable.

**Figure 5:**
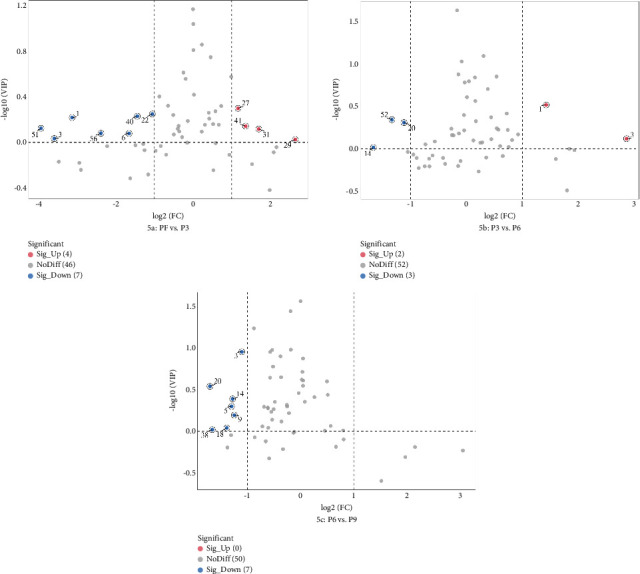
The Volcano plot of the different VOCs of PF vs F3 (a), F3 vs F6 (b), and F6 vs F9 (c).

**Figure 6:**
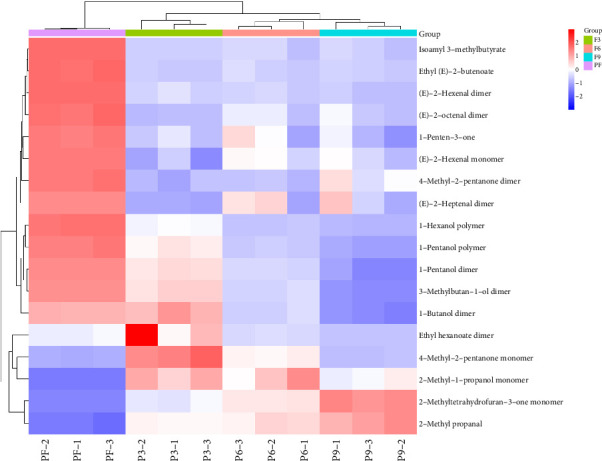
The heatmap of 18 VOCs present in different production stages of steaming *Polygonatum cyrtonema* Hua.

In addition, according to the principle of VIP > 1 and fold change ≥ 2 or ≤ 0.5 for the screening of the different VOCs, 11 differences were observed between PF and P3 (Figure 5(a)); 4 VOCs of 2-methyl propanal, 2-methyl-1-propanol, 2-methyltetrahydrofuran-3-one, and 4-methyl-2-pentanone showed upregulation; 7 VOCs of (E)-2-octenal, (E)-2-heptenal, (E)-2-hexenal, 1-penten-3-one, 4-methyl-2-pentanone, ethyl (E)-2-butenoate, and isoamyl 3-methylbutyrate showed downregulation. Five differences were observed between P3 and P6 (Figure 5(b)); (E)-2-octenal and (E)-2-heptenal showed upregulation; 1-hexanol polymer, 1-pentanol polymer, and ethyl hexanoate dimer showed downregulation. There are 7 differences between (E)-2-heptenal, (E)-2-hexenal, 1-butanol, 1-hexanol polymer, 1-pentanol, 1-pentanol polymer, and 3-methylbutan-1-ol showing downregulation between P6 and P9 (Figure 5(c)). The outcomes of gathering heatmap showed that the selected 18 VOCs were good for distinguishing the differences between fresh samples and different production stages of steaming *P. cyrtonema* Hua (Figure 6).

### 3.6. Nearest Neighbor Fingerprint Analysis of Different Production Stages of Steaming *P. cyrtonema* Hua

The NNFA can quickly compare the samples according to the intensity of the compounds in the selected evaluation area. It works by calculating the Euclidean distance between every two samples, and finding the “nearest neighbor” by searching for the minimum distance [26]. As shown in Figure S7 and Table S1, the Euclidean distance before and after steaming was far, indicating that the volatile components changed greatly before and after steaming, while the processed products with different steaming times were relatively concentrated, and a relatively small difference was observed between them.

## 4. Conclusions

In this study, the HS–GC–IMS analysis was used to study the VOCs of *P. cyrtonema* Hua before and after steaming and at different steaming times. The analysis of compound variations revealed distinct differences in the characteristic constituents of the samples before and after the steaming process. However, the distinctions among samples subjected to varying durations of steaming were relatively minor. A total of 92 different compounds were detected by GC–IMS, and 57 of these were identified, which included 17 aldehydes, 15 alcohols, 15 ketones, 5 esters, 3 furans, and 2 acids. Among them, compounds 1-hexanol and 1-pentanol exist in monomer, dimer, and polymer forms, and 15 compounds were found to exist in monomer and dimer forms. The contents of alcohol and ester were the highest in PF samples than others and decreased gradually with the increase in steaming times. The content of aldehydes and furans increased gradually with the increase in steaming times. During the continuous steaming process of *P. cyrtonema* Hua, with the increase in temperature and other factors, the ester compounds undergo a decomposition reaction, and alcohols may also be oxidized to aldehydes.

Further fingerprint analysis showed that the difference in VOCs in the samples during the steaming process was observed mainly in Regions A and B. In Region A, the content of VOCs before steaming was higher than that after steaming. Upon an increase in the frequency of steaming cycles, the concentrations of specific compounds exhibited a gradual decline, including 1-hexanol dimer (12), 1-hexanol monomer (13), and 3-methylbutan-1-ol dimer (38) (*p* < 0.05). Conversely, in Region B, an increase in the number of steaming cycles was associated with a significant rise in the levels of other compounds, such as 1-(2-furanyl)ethanone monomer (8), 2-furaldehyde (25), and 3-methyl butanal (36) (*p* < 0.05). In the hot processing, furfural compounds were generally produced by the Maillard reaction, such as isomerization of sugars and degradation under high temperature, mainly including 5-hydroxymethylfurfural (5-HMF), 2-furaldehyde (25), and 1-(2-furanyl)ethenone (8). 5-HMF and 2-furaldehyde are widely found in hot processed foods, such as coffee, beer, bread, vinegar, and tea [27]. In one study, furfural content in traditional Chinese fermented vinegar was positively correlated with free amino acids and sugars [28]. 3-methyl butanal (36) possesses a malty, fruity, cocoa-like odor and is widely used in fruit, chocolate, and coffee flavors. It was detected in thermally treated foods such as beef, chicken, chocolate, cocoa, coffee, bread, tea, and beer and is an important aroma compound derived from the Maillard reaction [29]. A reliable prediction model was established through OPLS-DA, and 18 markers (VIP > 1) were picked out for characterizing the fresh and processed samples three after steaming. In addition, the results of PCA and NNFA analysis showed that *P. cyrtonema* Hua before (PF) and after steaming (P3, P6, and P9) was distinct.

Therefore, HS–GC–IMS and chemometrics were used to characterize the VOCs at different production stages. This method has great application prospects, as it can quickly detect the flavor difference in the process of steaming and scientifically judge the degree of processing, to improve the quality and production efficiency of decoction pieces.

## Figures and Tables

**Table 1:** Headspace-gas chromatography-ion mobility spectrometry integration parameters of volatile compounds in different processing stages of steaming *Polygonatum cyrtonema* Hua.

NO.	Compound	CAS	Formula	MW	RI	Rt	Dt	Type	Mean peak area			
									PF	P3	P6	P9
1	(E)-2-Octenal dimer	C2548870	C_8_H_14_O	126.2	1441.3	1117.42	1.8215	Aldehyde	821.7	88.8	198.9	170.5
2	(E)-2-Octenal monomer	C2548870	C_8_H_14_O	126.2	1440.2	1114.712	1.34012	Aldehyde	5327.3	808.6	2103.7	1918.2
3	(E)-2-Heptenal dimer	C18829555	C_7_H_12_O	112.2	1327.9	880.217	1.66933	Aldehyde	1844.3	137.1	820.4	686.6
4	(E)-2-Heptenal monomer	C18829555	C_7_H_12_O	112.2	1327.3	879.244	1.26257	Aldehyde	2840.5	531.7	1659.2	1511.9
5	(E)-2-Hexenal dimer	C6728263	C_6_H_10_O	98.1	1232.4	718.858	1.51899	Aldehyde	7074.6	1087.1	932.3	478.8
6	(E)-2-Hexenal monomer	C6728263	C_6_H_10_O	98.1	1232.4	718.858	1.181	Aldehyde	2097.5	687.3	1158.8	999.6
7	1-(2-Furanyl)ethanone dimer	C1192627	C_6_H_6_O_2_	110.1	1542.8	1383.24	1.44346	Furan	4738.5	428.3	587.4	4511.8
8	1-(2-Furanyl)ethanone monomer	C1192627	C_6_H_6_O_2_	110.1	1546.4	1393.84	1.12052	Furan	679.9	1145.4	1913.6	7520.6
9	1-Butanol dimer	C71363	C_4_H_10_O	74.1	1164.2	601.412	1.38282	Alcohol	1926.9	1970.5	957.3	400.2
10	1-Butanol monomer	C71363	C_4_H_10_O	74.1	1164.4	601.859	1.18441	Alcohol	1698.7	2682.4	2264.5	1551.5
11	1-Hexanal	C66251	C_6_H_12_O	100.2	1101.2	487.886	1.55312	Aldehyde	13,649.1	5068.6	7900.0	6386.4
12	1-Hexanol dimer	C111273	C_6_H_14_O	102.2	1371.3	964.429	1.63795	Alcohol	8623.0	5359.3	2948.9	1238.4
13	1-Hexanol monomer	C111273	C_6_H_14_O	102.2	1374	969.844	1.3332	Alcohol	9581.4	8717.2	6787.2	4343.0
14	1-Hexanol polymer	C111273	C_6_H_14_O	102.2	1368.6	959.013	1.99292	Alcohol	3414.0	1188.1	402.1	170.4
15	1-Hydroxy-2-propanone dimer	C116096	C_3_H_6_O_2_	74.1	1314.1	855.093	1.23213	Ketone	1077.2	4209.1	5850.9	9023.3
16	1-Hydroxy-2-propanone monomer	C116096	C_3_H_6_O_2_	74.1	1317.5	861.249	1.05763	Ketone	3549.7	6815.4	6601.8	6772.0
17	1-Nonanal	C124196	C_9_H_18_O	142.2	1402.4	1029.585	1.47721	Aldehyde	1421.5	1154.8	1141.1	1244.7
18	1-Pentanol dimer	C71410	C_5_H_12_O	88.1	1263.3	767.874	1.5141	Alcohol	4910.2	3451.4	2228.4	1048.9
19	1-Pentanol monomer	C71410	C_5_H_12_O	88.1	1263.4	767.986	1.25412	Alcohol	4146.8	4210.2	3790.8	2641.1
20	1-Pentanol polymer	C71410	C_5_H_12_O	88.1	1263.3	767.874	1.81144	Alcohol	696.9	380.2	174.4	61.8
21	1-Penten-3-ol	C616251	C_5_H_10_O	86.1	1179.5	632.782	0.94303	Alcohol	2219.7	1547.8	1227.2	910.8
22	1-Penten-3-one	C1629589	C_5_H_8_O	84.1	1042.5	408.802	1.30535	Ketone	1282.1	640.5	740.1	572.9
23	1-Propanol	C71238	C_3_H_8_O	60.1	1057.8	427.944	1.25608	Alcohol	4944.9	2627.5	1444.3	975.3
24	2-Butanone	C78933	C_4_H_8_O	72.1	922.1	301.516	1.24558	Ketone	2487.1	7237.3	7869.9	8070.9
25	2-Furaldehyde	C98011	C_5_H_4_O_2_	96.1	1500	1264.102	1.33993	Aldehyde	827.9	3235.9	11,339.0	32,302.4
26	2-Heptanone	C110430	C_7_H_14_O	114.2	1194.2	662.722	1.62678	Ketone	5730.4	4330.6	2641.6	2028.6
27	2-Methyl propanal	C78842	C_4_H_8_O	72.1	812.9	247.679	1.28086	Aldehyde	375.3	883.3	961.9	1188.4
28	2-Methyl-1-propanol dimer	C78831	C_4_H_10_O	74.1	1113.3	507.867	1.36559	Alcohol	4280.1	6333.4	4122.2	2235.4
29	2-Methyl-1-propanol monomer	C78831	C_4_H_10_O	74.1	1110.5	503.205	1.17176	Alcohol	367.0	2155.2	2054.3	1497.8
30	2-Methyltetrahydrofuran-3-one dimer	C3188009	C_5_H_8_O_2_	100.1	1277.1	790.796	1.42652	Ketone	482.8	547.5	875.8	4098.6
31	2-Methyltetrahydrofuran-3-one monomer	C3188009	C_5_H_8_O_2_	100.1	1275.9	788.711	1.07578	Ketone	484.5	1651.9	2176.7	3183.7
32	2-Pentanone	C107879	C_5_H_10_O	86.1	1003.9	364.113	1.36415	Ketone	5728.9	2910.2	2813.8	3160.8
33	2-Pentyl furan	C3777693	C_9_H_14_O	138.2	1240.2	730.943	1.24933	Furan	5624.3	7909.4	9601.5	5126.4
34	3-Hydroxy-2-butanone dimer	C513860	C_4_H_8_O_2_	88.1	1299	828.414	1.33124	Ketone	8276.4	6578.9	5046.0	4110.0
35	3-Hydroxy-2-butanone monomer	C513860	C_4_H_8_O_2_	88.1	1299.6	829.44	1.06182	Ketone	3700.6	4700.1	4786.1	4543.7
36	3-Methyl butanal	C590863	C_5_H_10_O	86.1	936.4	311.389	1.40712	Aldehyde	2120.8	3077.3	3679.2	5013.5
37	3-Methyl-2-butenal	C107868	C_5_H_8_O	84.1	1214.3	691.734	1.09345	Aldehyde	166.8	237.5	387.0	500.9
38	3-Methylbutan-1-ol dimer	C123513	C_5_H_12_O	88.1	1220	700.169	1.49023	Alcohol	4404.8	3236.4	1837.5	601.4
39	3-Methylbutan-1-ol monomer	C123513	C_5_H_12_O	88.1	1220.6	701.125	1.24354	Alcohol	2280.3	3055.2	2620.6	1590.9
40	4-Methyl-2-pentanone dimer	C108101	C_6_H_12_O	100.2	1028.3	391.738	1.47861	Ketone	1085.7	387.7	424.3	634.1
41	4-Methyl-2-pentanone monomer	C108101	C_6_H_12_O	100.2	1034.5	399.121	1.17956	Ketone	649.8	1688.4	1113.5	734.8
42	Acetic acid dimer	C64197	C_2_H_4_O_2_	60.1	1510.1	1291.198	1.15704	Acid	4863.6	18,329.3	21,674.4	19,916.2
43	Acetic acid ethyl ester	C141786	C_4_H_8_O_2_	88.1	902.9	290.077	1.3316	Ester	13,138.8	5925.7	3650.2	3044.0
44	Acetic acid monomer	C64197	C_2_H_4_O_2_	60.1	1510.7	1293.005	1.05324	Acid	13,543.2	19,614.2	18,977.5	15,234.3
45	Acetone	C67641	C_3_H_6_O	58.1	837.9	258.773	1.11008	Ketone	22,554.3	20,489.7	18,302.5	16,858.1
46	Benzaldehyde	C100527	C_7_H_6_O	106.1	1555.4	1420.338	1.15192	Aldehyde	1946.5	2566.8	2834.5	2387.6
47	Butanal	C123728	C_4_H_8_O	72.1	892.5	284.848	1.28289	Aldehyde	1210.7	1334.8	1709.2	1641.1
48	Cyclopentanone dimer	C120923	C_5_H_8_O	84.1	1149.5	572.773	1.3338	Ketone	5166.8	2395.4	3410.4	5814.1
49	Cyclopentanone monomer	C120923	C_5_H_8_O	84.1	1153.3	579.932	1.11439	Ketone	871.6	745.3	2217.5	3582.7
50	Ethanol	C64175	C_2_H_6_O	46.1	959.6	328.04	1.14155	Alcohol	20,151.3	20,561.6	18,824.4	12,656.7
51	Ethyl (E)-2-butenoate	C623701	C_6_H_10_O_2_	114.1	1157	587.135	1.55941	Ester	1216.4	86.7	124.5	113.3
52	Ethyl hexanoate dimer	C123660	C_8_H_16_O_2_	144.2	1244	736.836	1.80154	Ester	158.2	383.0	121.3	83.9
53	Ethyl hexanoate monomer	C123660	C_8_H_16_O_2_	144.2	1245.5	739.197	1.34327	Ester	1101.7	1799.4	1000.8	645.8
54	Heptaldehyde dimer	C111717	C_7_H_14_O	114.2	1197.5	667.438	1.69635	Aldehyde	3358.7	1236.6	1176.0	1075.8
55	Heptaldehyde monomer	C111717	C_7_H_14_O	114.2	1198	668.088	1.33497	Aldehyde	1532.4	1032.3	1407.4	1535.3
56	Isoamyl 3-methylbutyrate	C659701	C_10_H_20_O_2_	172.3	1305.4	839.635	1.46361	Ester	1705.2	325.4	350.3	333.0
57	Propanal	C123386	C_3_H_6_O	58.1	798.8	241.608	1.14216	Aldehyde	6322.4	5175.9	5973.9	6156.4

## Data Availability

The data that support the findings of this study are available from the corresponding author upon reasonable request.

## Supporting Information

Supporting Information 1 Figure S1-1: The 2D spectrum of different production stages of steaming *Polygonatum cyrtonema* Hua (PF, P3, P6, and P9) analyzed by HS–GC–IMS.

Supporting Information 2 Figure S1-2: Differences in the 2D spectrum of different production stages of steaming *Polygonatum cyrtonema* Hua (PF, P3, P6, and P9).

Supporting Information 3 Figure S2: Three-dimensional pie chart of the number of compound types.

Supporting Information 4 Figure S3: Three-dimensional histogram of the percentage of compound types.

Supporting Information 5 Figure S4: PCA analysis of different production stages of steaming *Polygonatum cyrtonema* Hua.

Supporting Information 6 Figure S5: PLS-DA analysis of different production stages of steaming *Polygonatum cyrtonema* Hua.

Supporting Information 7 Figure S6: The OPLS-DA scatter plot (a) and cross-validation plot by 200 permutation tests (b) of PF vs. P3, P3 vs. P6, and P6 vs. P9.

Supporting Information 8 Figure S7: Nearest neighbor fingerprint analysis (NNFA) analysis of different production stages of steaming *Polygonatum cyrtonema* Hua.

Supporting Information 9 Table S1: The Euclidean distance of different production stages of steaming *Polygonatum cyrtonema* Hua.
