# Supplementary material for: HS–GC–IMS Coupled With Chemometrics Analyzes Volatile Aroma Compounds in Steamed Polygonatum cyrtonema Hua at Different Production Stages
Source: J Anal Methods Chem. 2025 Mar 10;2025:5592877. doi: 10.1155/jamc/5592877 (PMC11986191; doi:10.1155/jamc/5592877)
Supplement: Supporting Information 1 — Figure S1-1: The 2D spectrum of different production stages of steaming Polygonatum cyrtonema Hua (PF, P3, P6, and P9) analyzed by HS–GC–IMS. [file 5592877.f1.docx]

Supplementary material

**HS-GC-IMS coupled with chemometrics analyzes volatile aroma compounds in steamed *Polygonatum cyrtonema* Hua at different production stages**

**
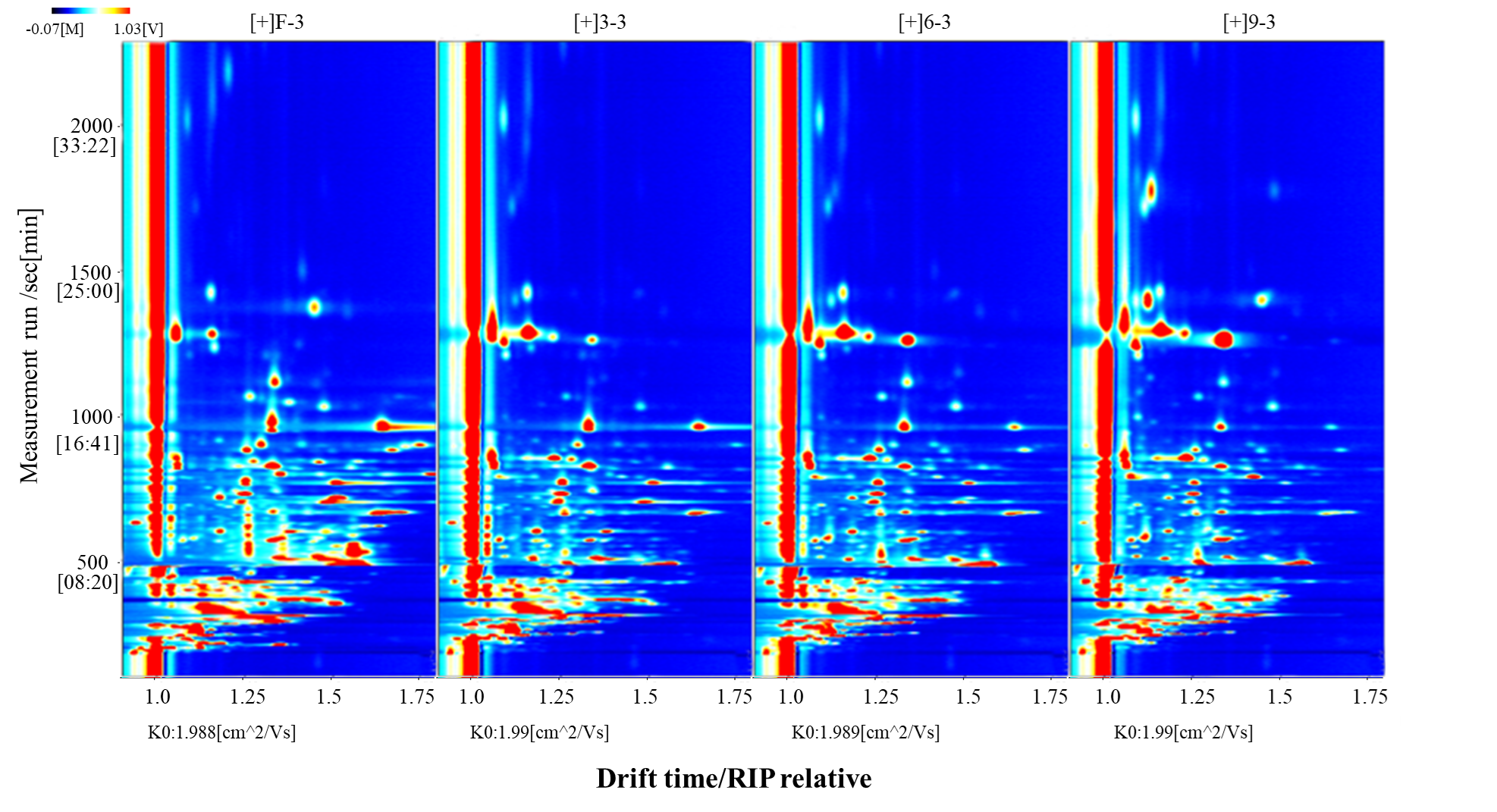
**

**Fig. S1-1 The 2D spectrum of different production stages of steaming *Polygonatum cyrtonema* Hua (PF, P3, P6, and P9) analyzed by headspace-gas chromatography-ion mobility spectrometry**
