# Supplementary material for: HS–GC–IMS Coupled With Chemometrics Analyzes Volatile Aroma Compounds in Steamed Polygonatum cyrtonema Hua at Different Production Stages
Source: J Anal Methods Chem. 2025 Mar 10;2025:5592877. doi: 10.1155/jamc/5592877 (PMC11986191; doi:10.1155/jamc/5592877)
Supplement: Supporting Information 2 — Figure S1-2: Differences in the 2D spectrum of different production stages of steaming Polygonatum cyrtonema Hua (PF, P3, P6, and P9). [file 5592877.f2.docx]

Supplementary material

**HS-GC-IMS coupled with chemometrics analyzes volatile aroma compounds in steamed *Polygonatum cyrtonema* Hua at different production stages**


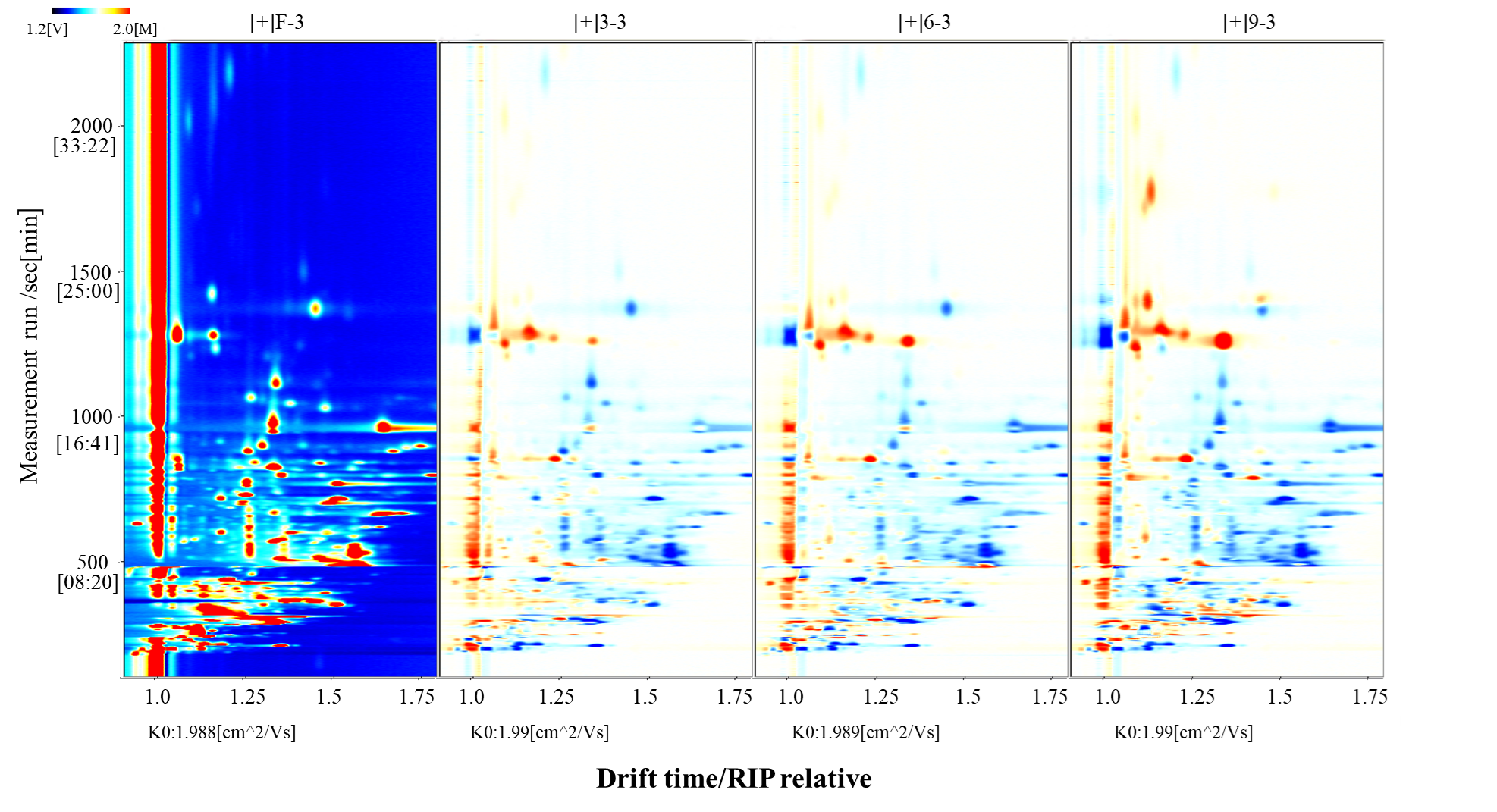


**Fig. S1-2 Differences in the 2D spectrum of different production stages of steaming *Polygonatum cyrtonema* Hua (PF, P3, P6, and P9)**
