# Supplementary material for: HS–GC–IMS Coupled With Chemometrics Analyzes Volatile Aroma Compounds in Steamed Polygonatum cyrtonema Hua at Different Production Stages
Source: J Anal Methods Chem. 2025 Mar 10;2025:5592877. doi: 10.1155/jamc/5592877 (PMC11986191; doi:10.1155/jamc/5592877)
Supplement: Supporting Information 7 — Figure S6: The OPLS-DA scatter plot (a) and cross-validation plot by 200 permutation tests (b) of PF vs. P3, P3 vs. P6, and P6 vs. P9. [file 5592877.f7.docx]

Supplementary material

**HS-GC-IMS coupled with chemometrics analyzes volatile aroma compounds in steamed *Polygonatum cyrtonema* Hua at different production stages**

**Fig. S6 The OPLS-DA scatter plot (a) and Cross-validation plot by 200 permutation tests (b) of PF vs. P3, P3 vs. P6, and P6 vs. P9**
