# Supplementary material for: HS–GC–IMS Coupled With Chemometrics Analyzes Volatile Aroma Compounds in Steamed Polygonatum cyrtonema Hua at Different Production Stages
Source: J Anal Methods Chem. 2025 Mar 10;2025:5592877. doi: 10.1155/jamc/5592877 (PMC11986191; doi:10.1155/jamc/5592877)
Supplement: Supporting Information 8 — Figure S7: Nearest neighbor fingerprint analysis (NNFA) analysis of different production stages of steaming Polygonatum cyrtonema Hua. [file 5592877.f8.docx]

Supplementary material

**HS-GC-IMS coupled with chemometrics analyzes volatile aroma compounds in steamed *Polygonatum cyrtonema* Hua at different production stages**


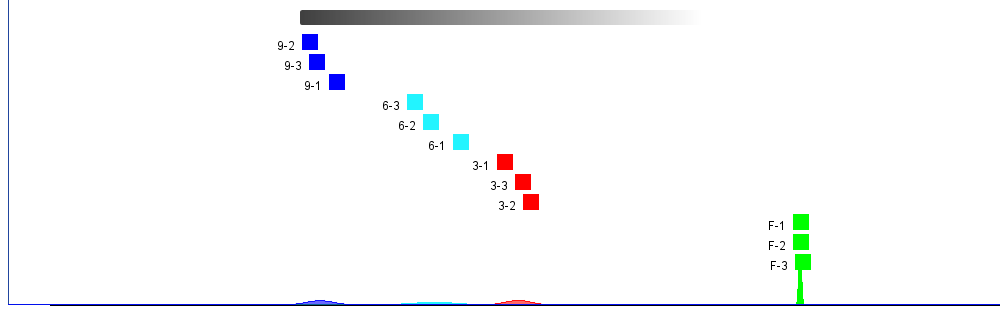
**Fig. S7 Nearest neighbor Fingerprint analysis (NNFA) analysis of different production stages of steaming *Polygonatum cyrtonema* Hua**
