# Supplementary material for: HS–GC–IMS Coupled With Chemometrics Analyzes Volatile Aroma Compounds in Steamed Polygonatum cyrtonema Hua at Different Production Stages
Source: J Anal Methods Chem. 2025 Mar 10;2025:5592877. doi: 10.1155/jamc/5592877 (PMC11986191; doi:10.1155/jamc/5592877)
Supplement: Supporting Information 9 — Table S1: The Euclidean distance of different production stages of steaming Polygonatum cyrtonema Hua. [file 5592877.f9.docx]

Supplementary material

**HS-GC-IMS coupled with chemometrics analyzes volatile aroma compounds in steamed *Polygonatum cyrtonema* Hua at different production stages**

**Table S1 The Euclidean Distance of different** **production stages of steaming *Polygonatum cyrtonema* Hua**

| **Samp.** | **Euclidean Distance** | | | | | | | | | | | |
| --- | --- | --- | --- | --- | --- | --- | --- | --- | --- | --- | --- | --- |
|  | **PF-1** | **PF-2** | **PF-3** | **P3-1** | **P3-2** | **P3-3** | **P6-1** | **P6-2** | **P6-3** | **P9-1** | **P9-2** | **P9-3** |
| **PF-1** | 0 | 55059 | 394802 | 14353397 | 14921672 | 15274913 | 19524319 | 16284783 | 15611693 | 21323889 | 25284204 | 23676154 |
| **PF-2** | 55059 | 0 | 158872 | 14786454 | 15388337 | 15822288 | 20187088 | 16702232 | 16037823 | 21775620 | 26016409 | 24323852 |
| **PF-3** | 394802 | 158872 | 0 | 15521903 | 16161860 | 16740754 | 21222580 | 17347407 | 16723842 | 22443193 | 27121932 | 25306566 |
| **P3-1** | 14353397 | 14786454 | 15521903 | 0 | 1083843 | 1031488 | 2440627 | 3340694 | 4021241 | 11403128 | 11588151 | 10876008 |
| **P3-2** | 14921672 | 15388337 | 16161860 | 1083843 | 0 | 481725 | 2969803 | 4106857 | 4529862 | 11766016 | 11087779 | 10474302 |
| **P3-3** | 15274913 | 15822288 | 16740754 | 1031488 | 481725 | 0 | 2764364 | 4358896 | 4608120 | 11893595 | 10813081 | 10417682 |
| **P6-1** | 19524319 | 20187088 | 21222580 | 2440627 | 2969803 | 2764364 | 0 | 1657796 | 2903550 | 7362914 | 6369392 | 6317466 |
| **P6-2** | 16284783 | 16702232 | 17347407 | 3340694 | 4106857 | 4358896 | 1657796 | 0 | 926863 | 5013411 | 6888990 | 5858932 |
| **P6-3** | 15611693 | 16037823 | 16723842 | 4021241 | 4529862 | 4608120 | 2903550 | 926863 | 0 | 4335774 | 6217326 | 4873481 |
| **P9-1** | 21323889 | 21775620 | 22443193 | 11403128 | 11766016 | 11893595 | 7362914 | 5013411 | 4335774 | 0 | 1737324 | 1115266 |
| **P9-2** | 25284204 | 26016409 | 27121932 | 11588151 | 11087779 | 10813081 | 6369392 | 6888990 | 6217326 | 1737324 | 0 | 425795 |
| **P9-3** | 23676154 | 24323852 | 25306566 | 10876008 | 10474302 | 10417682 | 6317466 | 5858932 | 4873481 | 1115266 | 425795 | 0 |
